# Elements of time and place: manganese and barium in shark vertebrae reflect age and upwelling histories

**DOI:** 10.1098/rspb.2018.1760

**Published:** 2018-11-07

**Authors:** John A. Mohan, Nathan R. Miller, Sharon Z. Herzka, Oscar Sosa-Nishizaki, Suzanne Kohin, Heidi Dewar, Michael Kinney, Owyn Snodgrass, R. J. David Wells

**Affiliations:** 1 Department of Marine Biology, Texas A&M University at Galveston, 1001 Texas Clipper Road, Galveston, TX 77553, USA; 2 Jackson School of Geosciences, The University of Texas at Austin, 2275 Speedway Stop C9000, Austin, TX 78712, USA; 3 Departamento de Oceanografía Biológica, Centro de Investigación Científica y de Educación Superior de Ensenada (CICESE), 3918 Carretera Tijuana –Ensenada, Ensenada, Baja California 22860, Mexico; 4 Southwest Fisheries Science Center, National Marine Fisheries Service, 8901 La Jolla Shores Dr, La Jolla, CA 92037, USA; 5 Ocean Associates, Southwest Fisheries Science Center, National Marine Fisheries Service, 8901 La Jolla Shores Dr, La Jolla, CA 92037, USA; 6 Department of Wildlife and Fisheries Sciences, Texas A&M University, College Station, TX 77843, USA

**Keywords:** shark, vertebral chemistry, barium, manganese, age, upwelling

## Abstract

As upper-level predators, sharks are important for maintaining marine food web structure, but populations are threatened by fishery exploitation. Sustainable management of shark populations requires improved understanding of migration patterns and population demographics, which has traditionally been sought through physical and/or electronic tagging studies. The application of natural tags such as elemental variations in mineralized band pairs of elasmobranch vertebrae cartilage could also reveal endogenous and exogenous processes experienced by sharks throughout their life histories. Here, elemental profiles were characterized in vertebrae encompassing complete life histories (birth-to-death) of shortfin mako (*Isurus oxyrinchus*), common thresher (*Alopias vulpinus*) and blue shark (*Prionace glauca*) of known tag and recapture locations in the eastern North Pacific Ocean. All sharks were injected with oxytetracycline at initial capture, released and subsequently recaptured, with individual liberty times ranging from 215 days to 6 years. Vertebral band pairs forming over the liberty intervals were verified by counting the number of band pairs deposited since the oxytetracycline band. Regular oscillations in vertebrae manganese (Mn) content corresponded well with the number of validated band pairs, suggesting that Mn variation could be used to age sharks. Increases in vertebrae barium concentration were correlated with times when individuals occupied areas with high coastal upwelling indices, the timing and spatial intensity of which varied from year to year. Interspecific relationships were probably influenced by behavioural differences in horizontal and vertical habitat use, feeding habits and thermoregulatory physiology. These results indicate that vertebral sclerochronology has the potential to advance our knowledge of elasmobranch life history including age and growth estimation and environmental reconstruction.

## 1. Background

Most elasmobranchs grow slowly, mature late, and have low fecundity and (normally) high longevity, in comparison with most exploited teleost species, all of which magnify the impact of fishing exploitation on their standing stocks [1]. For regional management and conservation of mobile species, it is essential to understand population productivity [2], dynamics of migration patterns, site fidelity, natal homing and connectivity within and among populations [3]. Highly migratory sharks transit through multinational waters where regulation is complicated and sharks are incidentally caught as by-catch in commerical longline, trawl, purse seine fisheries targeting other species and targeted in recreational fisheries [4]. In international fisheries, they are often not reported in catch statistics [5]. In addition to reliable catch statistics, accurate age and growth parameter estimates are particularly critical variables in stock assessment models, which are used to assess shark population demographics and setting target exploitation rates [1,6]. Incorrect age estimates can lead to erroneous conclusions regarding growth rates and population size, with severe consequences for shark population conservation [6].

Elasmobranchs are the only vertebrate group with non-bone cartilage skeletons reinforced with apatite [7]. Vertebrae columns of elasmobranchs grow radially and consist of calcified cartilage, which deposits calcium phosphate appositionally on an organic matrix [8]. Variations in calcium phosphate deposition result in the radial accretion of ‘band pairs’ that are distinguishable by optical properties due to different ratios of organic matrix to mineral content [9]. For most elasmobranch species, during fast summer growth, wider organic-rich hypomineralized zones are formed, whereas during slow growth, narrow hypermineralized zones are deposited, forming a band pair [9,10]. If the periodicity of band pair deposition is known or validated, then band pair counts can reveal age, since there is no resorption or remodelling of vertebral cartilage [11,12]. Several methods have been used to determine band pair periodicity in various species of elasmobranchs, including tag-recapture, marginal edge analysis, bomb radiocarbon and chemical tagging with oxytetracycline (OTC) [13]. Some of the limitations of these validation methods include the small sample sizes of recaptured sharks and that bomb radiocarbon techniques require that sharks were alive in the 1950s and 1960s, during and post-atomic bomb testing [14]. In addition, overall, age and growth studies using hard parts remain challenged by the difficulties of consistently and objectively identifying band pairs, and obtaining adequate sample sizes across the species size and geographical range.

To advance studies into age and growth as well as movement dynamics, researchers have examined the chemical composition of vertebrae. Although several studies have explored elemental variations (e.g. Ca, P, Sr) in vertebrae as they relate to band pair deposition [15–20], different species vary in their band pair elemental patterns. An early study of vertebrae band pair strontium (Sr) (in dogfish *Squalus acanthias* [18]) suggested Sr concentration was related to temperature and growth rate. Incorporation of other elements, such as Ba [16,21] and U [16], in addition to Sr [18,20,22,23] also appear to be mediated by the environment. Thus, vertebral band pair chemistry can reflect the ambient water mass or environmental conditions that a shark inhabited [21]. This interpretation has been widely used to assess movements across salinity gradients over ontogeny [22,23], geographical origin [24] and population structure [25–27]. While such studies assume element concentrations in vertebrae reflect ambient concentrations, the accuracy and precision of these interpretations remain tentative because the biochemical mechanisms of vertebrae mineralization are unknown [7] and few controlled laboratory experiments have been conducted to evaluate uptake of dissolved elements from water into vertebrae cartilage [21]. It is extremely difficult to conduct controlled causal experiments with large-bodied animals, but field studies using mark–recapture provide a viable alternative.

Accurate interpretation of elemental patterns requires validation studies, and no studies to date have explored elemental patterns in OTC age-validated sharks with known capture locations in natural conditions. Specimens for this study were provided from previous OTC age-validation studies of three federally managed shark species in the North Pacific Ocean's eastern boundary current, a highly productive system driven by coastal upwelling. The three species exhibit contrasting movement patterns: the shortfin mako *Isurus oxyrinchus*, which occupies coastal and oceanic habitats [28,29]; common threshers *Alopias vulpinus*, which primarily occupy coastal habitats [30] and blue sharks *Prionace glauca*, which are predominantly oceanic [31,32]. All three species interact with fisheries and are either targeted or taken incidentally. Effective management/stewardship of shark populations requires accurate sampling tools to improve understanding of migratory behaviour and population dynamics. This study explores chemical patterns in age-validated shark vertebrae with known capture–recapture locations, employing a natural based experiment to directly relate elemental variation to band pair deposition and environmental conditions in the southern California–Baja Mexico coastal upwelling ecosystem. The research objectives of this study were (1) to establish birth-to-death vertebral elemental profiles in species with contrasting movement patterns and habitat use and (2) to compare the documented vertebral elemental signatures with chemical oceanographic characteristics of tagging and recapture locations within the coastal upwelling system.

## 2. Methods

### (a) Shark tagging and recapture

**Figure 1.**
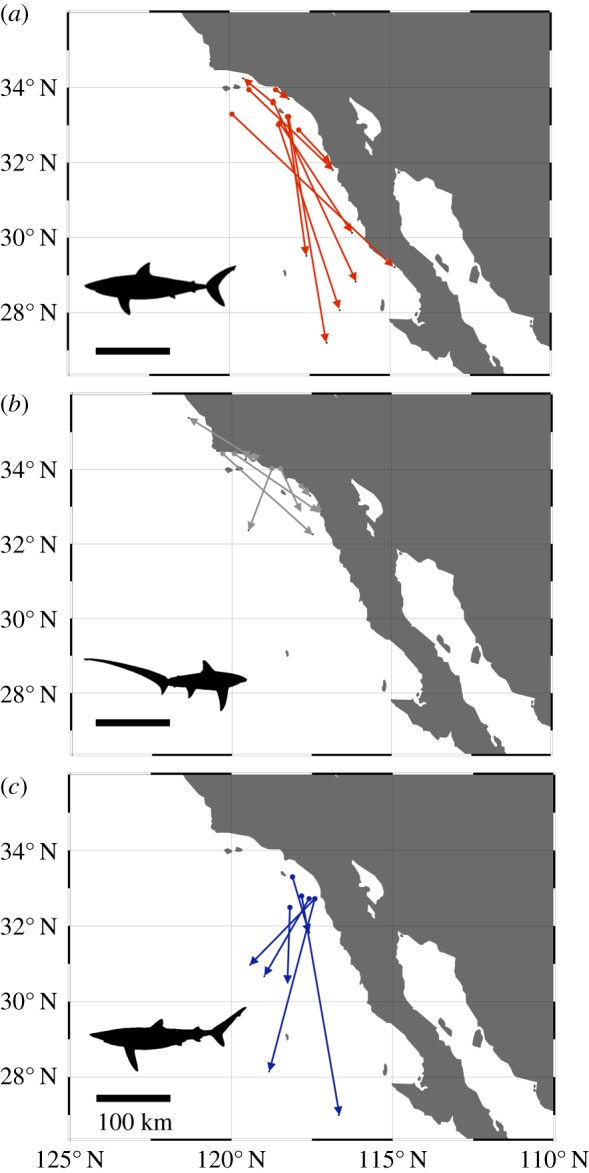
Map of shark capture and OTC tagging (circle) and recapture (arrow) locations for (*a*) shortfin mako (red), (*b*) common thresher (grey) and (*c*) blue sharks (blue) along the southern California coast, USA and the Baja California Peninsula, Mexico. See electronic supplementary material, table S1 for dates and duration between capture and recapture. (Online version in colour.)

Details of shark tagging with OTC and recapture can be found in [28,33] for shortfin mako, [34] for common thresher shark and [31] for blue shark. In brief, sharks were captured using a pelagic longline, brought onto a tagging cradle, and given an intraperitoneal injection of OTC at a dose of 25 mg kg^−1^ body mass, tagged externally with dart/roto tags containing recapture information, and then released. All OTC tagging was conducted in the summer months (June–September) and recaptures occurred from the spring throughout the autumn (see electronic supplementary material, table S1). Shortfin mako were recaptured in both coastal and oceanic environments, common thresher were primarily recaptured in coastal habitats and blue shark were mostly recaptured in oceanic locations (figure 1). Vertebrae from 10 shortfin mako, 10 common thresher and six blue shark were included in this study (see electronic supplementary material, table S1). Time at liberty varied for each species, with shortfin mako ranging from 277 to 2196 d, common thresher ranging from 267 to 1385 d and blue shark ranging from 215 to 587 d. Only one adult shortfin mako, that was at liberty for 6 years, was included in the study (A039494 [33]); all other sharks were juveniles or sub-adults. Annual deposition of band pairs has been validated in common thresher [34,35] and blue shark [31]; however, shortfin mako exhibit bi-annual deposition of band pairs when young, but transition to single band pair deposition at an unknown age [33].

### (b) Vertebrae preparation and analysis

Vertebrae centra were either stored dry, frozen or in ethanol, as previous experiments have demonstrated minimal influence of these storage methods on primary elemental composition [36]. Vertebrae centra were cut using a low-speed diamond blade saw along central longitudinal planes to obtain approximately 2 mm thick central ‘bowtie’ sections. Sectioned ‘bowties’ were affixed to petrographic slides using thermoplastic cement (Crystalbond). Elemental concentrations in the direction of radial (outward) growth were quantified using a New Wave Research UP 193-FX fast excimer (193 nm wavelength, 4–6 ns pulse width) laser system coupled to an Agilent 7500ce inductively coupled mass spectrometer at the University of Texas at Austin. The laser system is equipped with a large format two-volume laser cell, which accommodated all shark vertebrae mounts and standards in a single loading with analysis occurring over 48 h. We first established a two-dimensional chemical map over a 1.5 × 4.5 mm area of shortfin mako sample A038423 that encompassed the complete corpus calcareum, from the vertebral focus to the marginal edge. Analytical parameters were 90% laser power, 20 Hz repetition rate, 15 × 15 µm^2^ aperture, 150 µm s^−1^ scan rate and a He cell flow of 800 ml min^−1^. Prior to analysis, the sample was pre-ablated at 70% power, 20 Hz, using a 150 µm spot scanning at 150 µm s^−1^. The imaged area involved 101 line traverses, spaced 15 µm apart, with standards (NIST 612) analysed each hour. Single line transects were completed for all other vertebrae samples. Laser ablation parameters optimized from test ablations were 60% laser power, 10 Hz repetition rate, 100 µm spot, 10 µm s^−1^ scan rate and a He cell flow of 800 ml min^−1^. Prior to analysis, sample and standard traverses were pre-ablated at 60% power using a 125 µm spot scanning at 100 µm s^−1^, to remove potential surface contamination. Laser transect analyses were bracketed every hour by standard measurements (USGS MAPS-4, MACS-3, and NIST 612; measured in triplicate for 60 s). Laser energy densities over the analytical sessions averaged 3.07 ± 0.08 J cm^−2^ for line traverses. The quadrupole time-resolved method measured six masses using integration times of 10 ms (^24^Mg, ^43^Ca, ^55^Mn, ^88^Sr) and 20 ms (^66^Zn, ^138^Ba). Time-resolved intensities were converted to concentration (ppm) equivalents using Iolite software (Univ. Melbourne [37]), with ^43^Ca as the internal standard and a Ca index value of 35 weight % ([38], table III). Concentration (in ppm) was also expressed as molar ratios to calcium (in μmol mol^−1^), to make data units comparable to previous studies. Baselines were determined from 30 s gas blank intervals measured while the laser was off and all masses were scanned by the quadrupole. USGS MAPS-4 was used as the primary reference standard. Analyte recoveries for secondary standards MACS-3 and NIST 612, respectively, averaged 106 ± 1.1% and 112 ± 0.1% (*n* = 18; versus GeoREM preferred values; http://georem.mpch-mainz.gwdg.de). Image data were processed using Iolite [37]. Digital images of each vertebrae were acquired on a stereoscope mounted with a camera and measurements recorded in ImageJ software. Distance (in μm) along each vertebrae were measured in triplicate and averaged for: (i) initial focal point ablation to birth band; (ii) from birth band to edge; (iii) from OTC mark to edge; and (iv) from focal point to edge of vertebrae. The elemental transect between the visible OTC mark and vertebrae outer edge, represents the capture–recapture migration time within the coastal region.

### (c) Data analysis

Elemental transect data were smoothed using 7-point median followed by 7-point moving average to remove high-frequency noise. To make comparisons of Mn : Ca data among species, concentration data were further smoothed using a second-order 50 nearest-neighbour smoothing function, and values were then expressed as a proportion of the maximum value in individual time series to normalize variable concentrations among species and plotted for each shark (see the electronic supplementary material, figure S1). Normalized Mn peaks were counted by three independent readers and reported as mean ± s.e. Vertebral Ba : Ca concentrations were averaged across distances of 100 µm at the OTC mark (known capture) and at the vertebrae edge (known recapture). The average daily accretion rate for all juvenile shark vertebrae was 3.6 ± 1.2 µm d^−1^ (electronic supplementary material, table S1); thus, 100 µm of elemental data represents approximately one month (28 d) (electronic supplementary material, figure S1).

Coastal upwelling indices, which estimate the intensity of Ekman transport based on offshore wind measurements, were obtained from the Pacific Fisheries Environmental Laboratory website (http://pfeg.noaa.gov/products/PFEL/modeled/indices/PFELindices.html). Upwelling stations closest to the point of capture and recapture for individual shark specimens (specifically stations A: 36° N 122° W; B: 33° N 119° W; C: 30° N 119° W; and D: 27° N 116° W) were selected to obtain monthly upwelling indices. Upwelling indices for the month of capture and the previous month were summed, to account for lagged effects of upwelling on food webs and transfer of primary production across trophic levels. Linear regression analysis was performed to investigate how well band pair counts/validated age and upwelling indices (independent variables) predicted vertebral Mn : Ca and Ba : Ca (dependent variables), respectively.

## 3. Results

**Figure 2.**
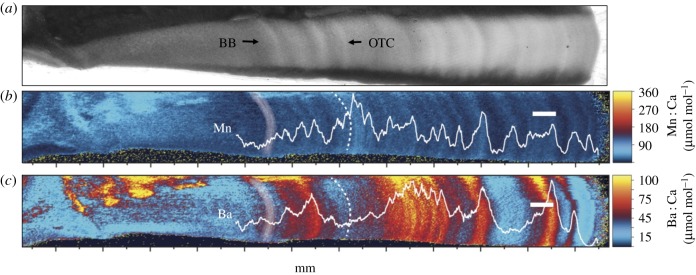
(*a*) Image of shortfin mako (ID: A038423) vertebrae under transmitted light, and (*b*,*c*) two-dimensional elemental map of (*b*) manganese and (*c*) barium variation. Birth band (BB) denoted in (*b*) and (*c*) with white shading and oxytetracycline band (OTC) denoted with dashed lines. Translucent zones (hypermineralized, slow growth) in a), matches with Mn decreases; opaque hypomineralized high protein zones matches with Mn peaks. White scale bar, 500 µm.

The two-dimensional elemental map of the single shortfin mako vertebrae corpus calcareum reveals that Mn and Ba concentrations oscillate between lower and higher concentrations, but at differing frequencies (figure 2). The other elements quantified did not exhibit consistent patterns and thus are not presented (but see electronic supplementary material, figure S2). The period of Mn spatial variation appears to correspond well with visible banding patterns (figure 2*a*,*b*). Barium patterns, however, are more variable, ranging from elevated values that span multiple years of growth, to lower values in other years (figure 2*c*). Comparison between the two-dimensional elemental map and a single laser transect along the centre of the corpus calcareum indicate that laser transects accurately track radial variability of Ba : Ca and Mn : Ca during vertebrae growth (figure 2), and that single transects sufficiently capture element patterns.

**Figure 3.**
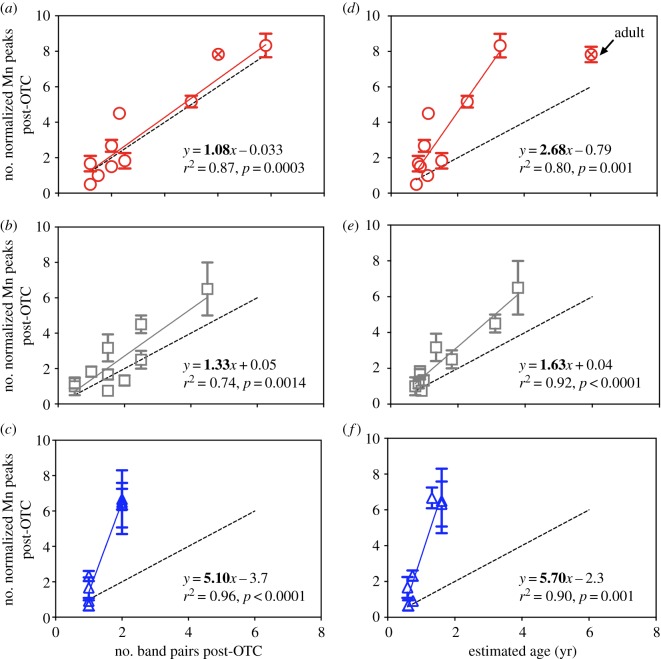
(*a*–*c*) Number of normalized Mn peaks post-OTC band versus number of band pairs post-OTC for (*a*) shortfin mako, (*b*) common thresher and (*c*) blue sharks. (*d*–*f*) Number of normalized Mn peaks post-OTC band versus validated age in years (yr) post-OTC band for (*d*) shortfin mako (*e*) common thresher and (*f*) blue sharks. Dashed black line = 1 : 1 relationship. The adult mako shark (circle with cross symbol) from [33] was not used in regression analysis. (Online version in colour.)

**Figure 4.**
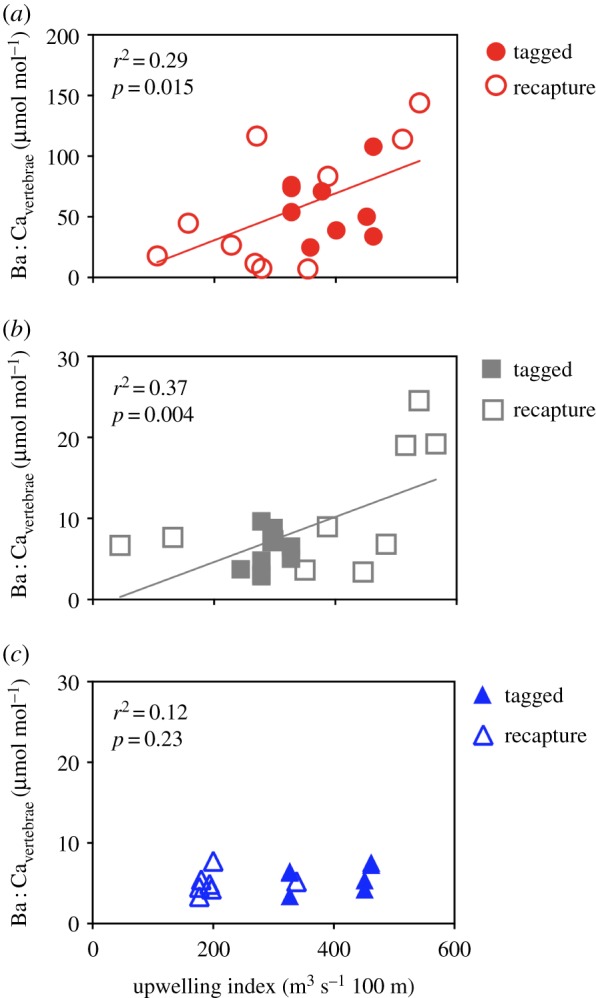
Relationship between cumulative monthly upwelling index (month of capture + previous month) at nearest station and Ba : Ca in vertebrae from known OTC tagging location (solid symbols) and known recapture location (open symbols) for (*a*) shortfin mako red circles), (*b*) common thresher (grey squares) and (*c*) blue shark (blue triangles). Solid coloured lines indicate linear regression equations. (Online version in colour.)

We next compared single laser transect profiles in Mn : Ca and Ba : Ca among individuals of other species. For Mn : Ca, we found that the number of smoothed and normalized post-OTC peaks consistently related to the number of band pairs and validated age for each species (figure 3). However, relationships were species-specific, with shortfin mako having 1.08 ± 0.16 Mn peaks per band pair (figure 3*a*) and 2.68 ± 0.50 Mn peaks yr^−1^ (figure 3*d*); common thresher having 1.33 ± 0.28 Mn peaks per band pair (figure 3*b*) and 1.63 ± 0.17 Mn peaks yr^−1^ (figure 3*e*); and blue shark having 5.10 ± 0.45 Mn peaks per band pair (figure 3*c*) and 5.70 ± 0.83 Mn peaks yr^−1^(figure 3*f*). A comparison of upwelling indices and the approximately 1 month around release and recapture when location was known revealed species-specific relationships (figure 4). Corresponding linear relationships between upwelling intensity and vertebral Ba : Ca were significant for shortfin mako (figure 4*a*; *y* = 0.193*x* – 7.9; *F*_1,18_ = 7.26; *p* = 0.015) and common thresher (figure 4*b*; *y* = 0.028*x* – 0.94 l; *F*_1,18_ = 10.74; *p* = 0.004), but not blue shark (figure 4*c*).

## 4. Discussion

Reliable interpretation of elemental patterns in shark vertebrae to characterize life histories requires validation studies. Towards that end, this study compared vertebral elemental time-series among individuals from three species of sharks from three different families, each with known age and known capture locations. We found evidence that Mn : Ca variation relates to band pair deposition and that Ba : Ca increases corresponds with the intensity of oceanic upwelling. These findings support the potential of Mn : Ca variation for estimating age and Ba : Ca concentrations, in some species, to reconstruct the relative intensity of upwelling in coastal and oceanic habitats, and more generally the potential of natural chemical tags for improving our understanding of population ecologies among elasmobranch species.

The uptake of trace elements in fish tissue and hard parts can occur via passage of dissolved ions from the water through the gill membrane or through diet and intestinal pathways; research to date suggests dietary uptake is dominant in elasmobranchs [39]. Metals in fish blood can occur as free hydrated species or may be bound in organic complexes or proteins [40], which can affect their incorporation into mineralized cartilage [38]. Alkaline earth metals (i.e. Mg, Ca, Sr, Ba) have similar ionic radii and +2 charge, which substitute for Ca during biomineralization of teleost otolith aragonite [41] and elasmobranch hydroxyapatite [21,38]. In precipitation of synthetic hydroxyapatite, Mn directly substitutes for Ca during formation [42]. Manganese concentrations have been found to be elevated in calcified tissues (vertebrae) compared to soft tissues in many species of shark, suggesting that Mn uptake and accumulation is related to calcium metabolism [43]. Although the pathway for Mn incorporation is not well understood, in shark vertebrae Mn variation appears to be coupled to band pair formation making it useful to age sharks.

### (a) Manganese reflects age

The strong relationship between Mn and age corresponds to regular oscillations in Mn concentration in hypo- and hypermineralized band pairs. Hypomineralized regions associated with fast growth during spring and summer seasons have notably higher Mn concentrations. Several mechanisms could account for elevated Mn levels in hypomineralized zones of vertebra band pairs. If Mn is primarily from the diet, then periods of high prey consumption and rapid growth should result in more Mn incorporation. Alternatively, higher Mn in hypomineralized zones could be related to higher organic matter/protein content in vertebral tissue relative to the mineralized fraction during periods of faster growth [44]. It is plausible that Mn is incorporated into both the organic and inorganic fractions of vertebral cartilage, but at different proportions, as found in vertebrate bone [45]. All three species displayed different relationships between band pair deposition and Mn peaks. Common thresher displayed 1.3 ± 0.28 Mn peak per band pair and about 1.6 ± 1.17 Mn peak per year that corroborates annual band pair deposition [34,35,46]. The shortfin mako exhibited 1.03 ± 0.16 Mn peak per band pair and 2.68 ± 0.5 Mn peaks per year, which closely matches the bi-annual deposition rate in juveniles from both verification [47] and validation studies [28]. However, the single adult mako that was tagged at 5 years old, and then recaptured 6 years later did display 1.3 Mn peak per year, supporting annual band pair deposition in adult makos [33,48,49]. Blue sharks showed about five Mn peaks per band pair (5.1 ± 0.45) and per year (5.7 ± 0.83), but this species exhibited shorter times at liberty (0.6–1.6 yr) compared to the threshers and makos. Determining Mn peaks in blue sharks may have been hindered by shorter times at liberty and the challenges of identifying partial band pairs. Perhaps juvenile blue shark growth rates are variable within a season, resulting in multiple Mn peaks within a single band pair, because annual deposition has been previously validated in blue sharks [31].

Although Mn varied in a predictable manner with true age, the mechanisms driving variable Mn–age relationships among species require further study. Differences in Mn–age relationships could also be due to species-specific calcification physiology [50], dietary preferences [51], or migration patterns and habitat use [5]. For example, telemetry studies of blue sharks have found seasonally variable depth preferences, perhaps due to feeding upon seasonally abundant prey associated with different depths [52]. A comparative study of diets among these three species from the California coast reported opportunistic feeding and diverse diets in makos and blue sharks feeding primarily on squid and fish, but dietary specialization in thresher sharks preferring small schooling fish [51]. Additionally, males and females were not equally represented among the species in this study and had to be examined together due to limited sample sizes, which may have induced variability in terms of physiology and behaviour between sexes or size classes. Using elemental profiles obtained from vertebrae could potentially be used to estimate and validate age, derive age–length relationships and estimate growth rates based on large numbers of samples, if the species-specific relationship between band pair deposition and elemental chemistry are well characterized. Although vertebrae sampling is necessarily lethal, it does not require recapture of individuals and could be applied to sharks that are difficult to tag and recapture, such as deep-sea species with limited life-history information [53]. Vertebral elemental analyses may offer an advantage over traditional age validation approaches, such as OTC tagging and recapture that are time consuming, expensive and limited by small sample sizes [1].

### (b) Barium reflects upwelling intensity

Oceanic upwelling transports deep, nutrient-rich (e.g. P, N, Si, Ba, Cd) cold water to the surface due to wind-driven Ekman transport [54]. Regions of upwelling exhibit increased Ba concentrations in surface waters [55], which may be incorporated in associated biominerals such as teleost fish otoliths [56] and coral skeletons [57,58]. That vertebral Ba levels over the approximately one month period examined in shortfin makos and common threshers correspond to the intensity of oceanic upwelling at known capture locations and dates along the California coast demonstrates the potential of shark vertebrae for proxying aspects of water mass chemistry. Barium patterns in the vertebrae of white sharks (*Carcharodon carcharias*) have been suggested to be derived from seasonal upwelling off South Africa [16].

Physiological controls of vertebral Ba variation in elasmobranchs are unknown with few controlled studies [21,22], but could be related to reduced sea surface temperature or increased dissolved [Ba] in seawater, both associated with upwelling conditions. Smith *et al*. [21] found the temperature and ambient dissolved element concentrations can influence vertebral chemistry. Using round stingrays *Urobatis halleri* held for 3.6–8 months in three temperatures (16°, 18°, 24°) and three dissolved barium concentrations (1×, 3×, 6× ambient), they found a negative relationship between temperature and barium incorporation and a significant positive relationship between vertebrae and water Ba : Ca. Cold upwelled waters with higher Ba : Ca concentrations, may have likewise influenced Ba uptake in the shortfin mako and common thresher vertebrae studied here. Consistent with this result, synthetic hydroxyapatite incorporates more Ba at lower temperatures [59]. Alternatively, if vertebrae Ba concentration is also influenced by dietary pathways, then sharks feeding in upwelling regions may incorporate elevated levels of Ba from ingested prey [60]. Juvenile threshers use highly productive continental shelf waters as nursery habitat and display a preference for the upper mixed layer (less than 20 m) where there is abundant northern anchovy *Engraulis mordax* prey [30]. In the California Current region, anchovy populations become more abundant during high upwelling years [61].

Based on validated vertebral-based growth rates, a distance of 100 µm represents approximately one month of time. Previous studies reporting elemental uptake rates of two to four weeks for elasmobranch vertebrae chemistry to equilibrate with water chemistry [21,22]. Thus, the time frames represented by the vertebrae Ba : Ca sampling and upwelling index match temporally, which may contribute to the statistically significant relationship between upwelling and barium in shortfin makos and common threshers.

The lack of a significant relationship between vertebrae Ba : Ca and upwelling intensity for blue sharks could result from a range of factors. Blue shark diet, habitat and physiology differs from shortfin makos and thresher sharks. Blue sharks spend more time in pelagic oceanic habitats [62], have a greater reliance on deep water prey associated with the deep scattering layer (DSL) [51,52], and use offshore nursery habitats. In comparison, shortfin mako and thresher shark spend more time in coastal habitats and the nursery habitat for both species is considered to be the coastal waters, including the continental shelf and edge where upwelling occurs [29,30]. The diets of mako and thresher sharks include organisms of the DSL but to a lesser extent than blue sharks, and at smaller sizes mako and thresher sharks forage on a range of species. Additionally, being ectotherms, blue sharks probably have the lowest metabolic rates of the three species, whereas mako and thresher sharks are regionally endothermic and use a heat-exchanging circulatory system that maintains muscle temperature above ambient water temperature [63,64]. A lower metabolic rate may influence rates of uptake and incorporation of Ba : Ca. These differences in diet, habitat and metabolic rates among species, likely explain the discrepancy between vertebrae barium and upwelling in blue sharks compared to shortfin makos and common threshers.

## 5. Conclusion

Elemental patterns in mineralized vertebral cartilage from individuals of three shark species, each with known capture–recapture (liberty) durations and locations are interpreted as proxies of age (Mn : Ca) and intensity of environmental upwelling (Ba : Ca). Patterns are species-specific, with differences likely related to varied habitats and thermal preferences (coastal upwelling zones versus pelagic habitat), metabolic rates (endothermic versus ectothermic) and foraging ecology. Only one sexually mature shortfin mako was analysed in this study and future work should include older sharks to characterize elemental chemistry across ontogeny and potential variations associated with reproduction and sexually dimorphic behaviour. Multi-decadal archives of shark vertebrae are available in historical collections that could be used to establish long-term climate biochronologies and investigate how oceanic regimes (e.g. El Nino, Pacific Decadal Oscillation) affect upwelling recorded in vertebral barium patterns and the consequences for growth, by measuring band-pair increment width (e.g. [65]). This information could be used to inform ecosystem-based management practices to better conserve predator populations.

## Supplementary Material

Figures S1 and S2

## Supplementary Material

Data

## Data accessibility

The dataset supporting this article is available in the electronic supplementary material.
