# Supplementary material for: Elements of time and place: manganese and barium in shark vertebrae reflect age and upwelling histories
Source: Proc Biol Sci. 2018 Nov 7;285(1890):20181760. doi: 10.1098/rspb.2018.1760 (PMC6235039; doi:10.1098/rspb.2018.1760)

Electronic Supplemental Material

Elements of time and place: manganese and barium in shark vertebrae reflect age and upwelling histories

Proceedings of the Royal Society Biology

DOI: 10.1098/rspb.2018.1760

John A. Mohan[1], Nathan R. Miller[2], Sharon. Z. Herzka[3], Oscar Sosa-Nishizaki[3], Suzanne Kohin[4], Heidi Dewar[4], Michael Kinney[5], Owyn Snodgrass[5], R.J. David Wells[1,6]

[1] Texas A&M University at Galveston, Department of Marine Biology, 1001 Texas Clipper Rd., Galveston, TX 77553, USA

[2] The University of Texas at Austin, Jackson School of Geosciences, 2275 Speedway Stop C9000, Austin, TX 78712, USA

[3] Departamento de Oceanografía Biológica, Centro de Investigación Científica y de, Educación Superior de Ensenada (CICESE), 3918 Carretera Tijuana –Ensenada, Ensenada, Baja California 22860, México

[4] Southwest Fisheries Science Center, National Marine Fisheries Service, 8901 La Jolla Shores Dr., La Jolla, CA 92037, USA

[5] Ocean Associates, Southwest Fisheries Science Center, National Marine Fisheries Service, 8901 La Jolla Shores Dr., La Jolla, CA 92037, USA

[6] Texas A&M University, Department of Wildlife & Fisheries Sciences, College Station, TX 77843, USA

Table 1. Summary statistics of each shark including shark identification (Id), time at liberty (days), tagging and recapture dates, size (FL=fork length), sex, and band pair counts post-OTC and post-birth band. One mako (A039494) was an adult; all other sharks were juveniles or sub-adults. One blue shark (^) had two vertebrae analyzed so the growth rate reported represents an average. ND: No data.

Species	Shark Id	Time at liberty	Tagging date	Recapture date	Tagging length (cm FL)	Recapture length (cm FL)	Sex	# band pairs after OTC mark	# band pairs post birth mark	Vertebrae growth rate (µm d^{-1})
mako	A039494	2196	6/26/08	7/1/14	194	217	M	6	15.5	0.98
mako	A038423	1198	7/9/05	10/19/08	91	157	M	7.8	11.5	4.60
mako	A039374	364	7/15/07	7/13/08	129	154	M	2	7	3.34
mako	A038734	836	7/5/04	10/19/06	106	172	M	5	9	4.28
mako	A039341	400	7/24/07	8/27/08	131	160	M	1.5	5.5	3.42
mako	A040374	298	8/15/09	6/9/10	100	ND	F	1.2	3.2	3.03
mako	A040354	407	8/13/09	9/24/10	98	137	M	2.3	3.8	4.79
mako	A039946	555	8/1/07	2/6/09	109	150	M	2.5	4.5	1.92
mako	A040302	277	8/8/09	5/12/10	89	103	M	1.2	3.5	3.09
mako	A040327	323	8/11/09	6/30/10	96	114	M	2	5	2.89
thresher	A039565	1134	9/5/07	10/13/10	113	165	F	2.5	3.5	2.57
thresher	A039063	1385	9/9/06	6/25/10	85	142	F	4.5	4.5	2.58
thresher	A079055	666	9/13/09	7/11/11	128	168	F	2.5	5	3.80
thresher	A079196	267	9/6/09	5/31/10	92	112	F	0.5	2	2.19
thresher	A039552	336	9/1/07	8/2/08	74	ND	M	1.5	2	3.96
thresher	A039611	304	9/8/07	7/8/08	96	137	F	0.5	1	2.77
thresher	A040684	314	9/19/08	7/30/09	116	137	F	1.5	3	3.25
thresher	A038148	494	9/13/06	1/20/08	108	128	M	1.5	2	3.35
thresher	A040854	357	9/5/09	8/28/10	104	120	F	2	2.5	6.35
thresher	A040898	319	9/5/09	7/21/10	103	108	M	1	2.5	5.51
blue^	A039329	587	7/22/07	2/28/09	86	123	F	2	3	5.48
blue	A039424	482	6/22/2008	10/17/09	73	98	M	2	3	5.17
blue	A040740	215	8/17/2009	3/20/10	82	ND	F	1	2	2.97
blue	A039872	292	6/20/2008	4/8/09	87	ND	F	1	2	3.37
blue	A040274	273	8/6/2009	5/6/10	83	123	F	1	1	2.60
blue	A040270	223	8/6/2009	3/17/10	88	90	M	1	2	2.16

Fig S1. a) Example of laser transect (red dashed line) across vertebrae band pairs (shark A038734) in transmitted (top) and fluorescent light (bottom) and calculation of growth rate between OTC mark (black box) and edge (black box) known locations; b) Mn:Ca and c) Ba:Ca versus laser distance, gray boxes denote birth band, OTC tag, and recapture, where 100 microns of material represents approximately 1 month; d) Mn:Ca data were smoothed and normalized to visually count Mn peaks (red numbers) between the OTC mark and the edge (at recapture).

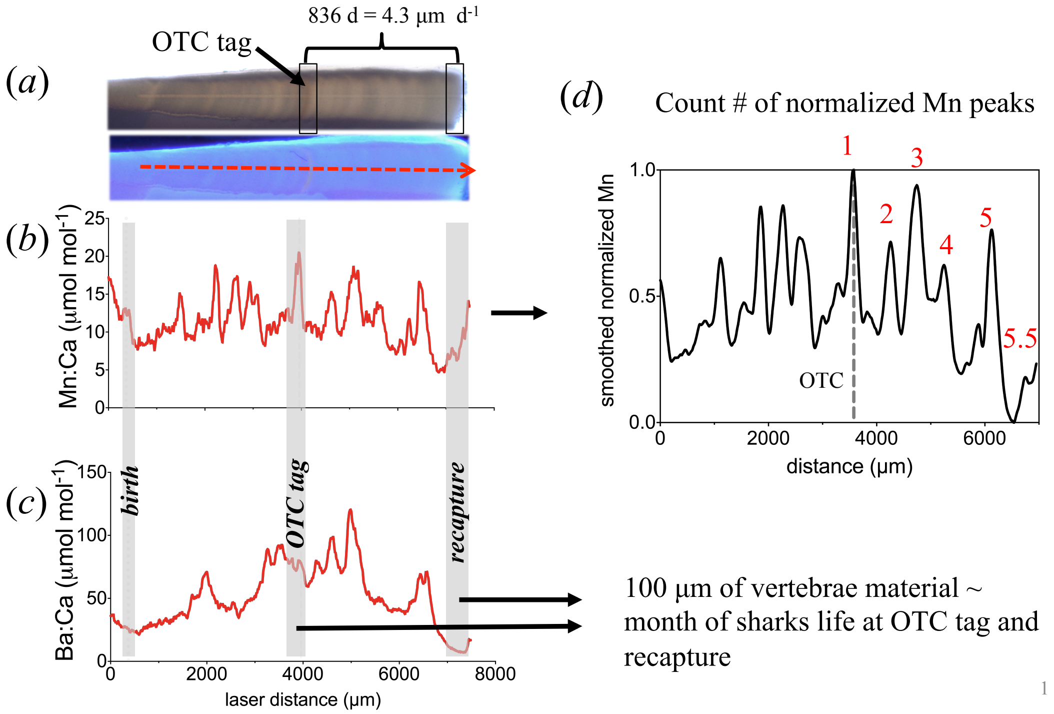

Fig S2. Elemental transect plots of internal standard calcium (Ca in CPS) (first column); Mn:Ca (second column); Sr:Ca (third column) and Ba:Ca (forth column) for all individual sharks (rows).

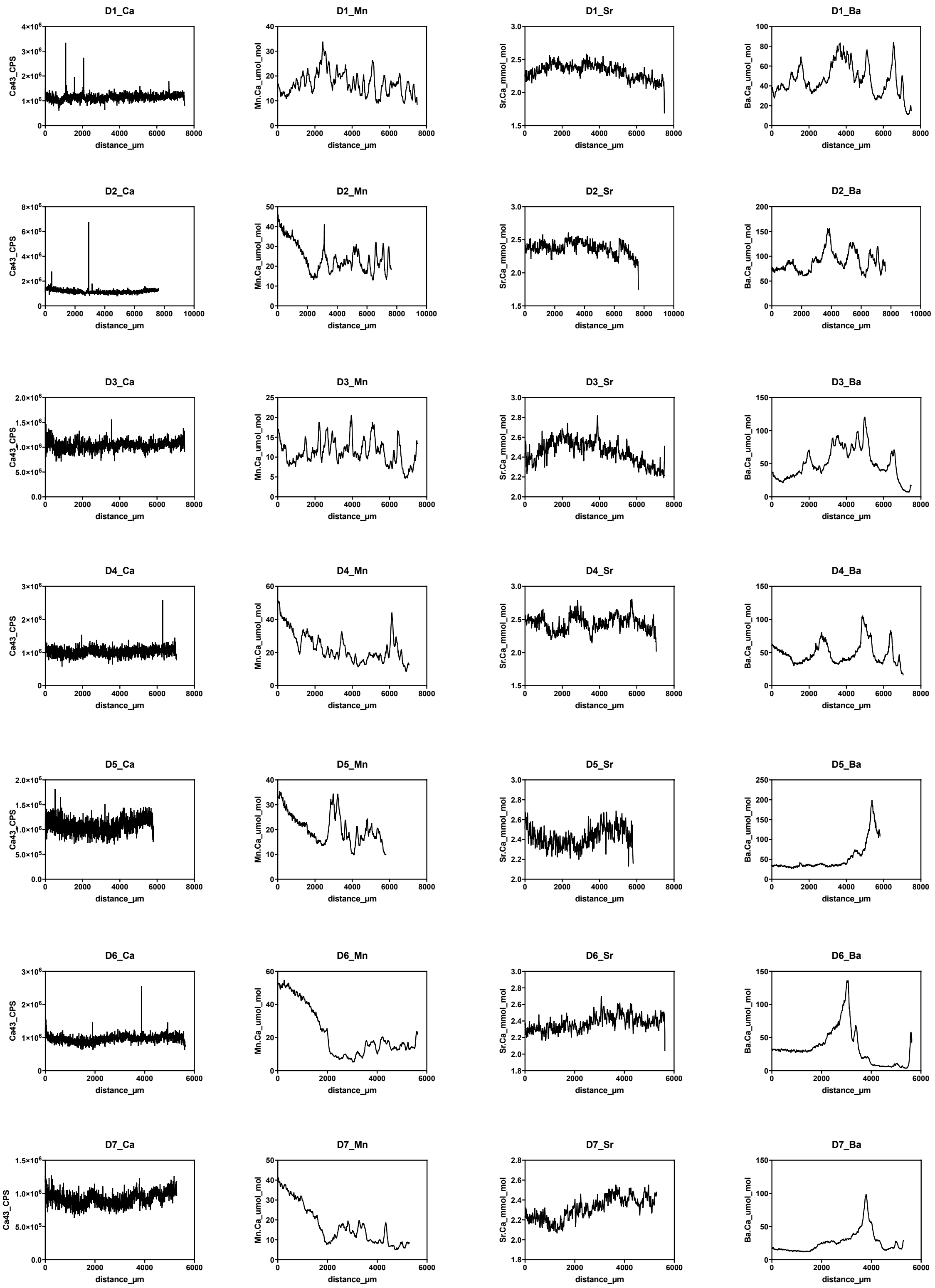

Fig S2. Continued

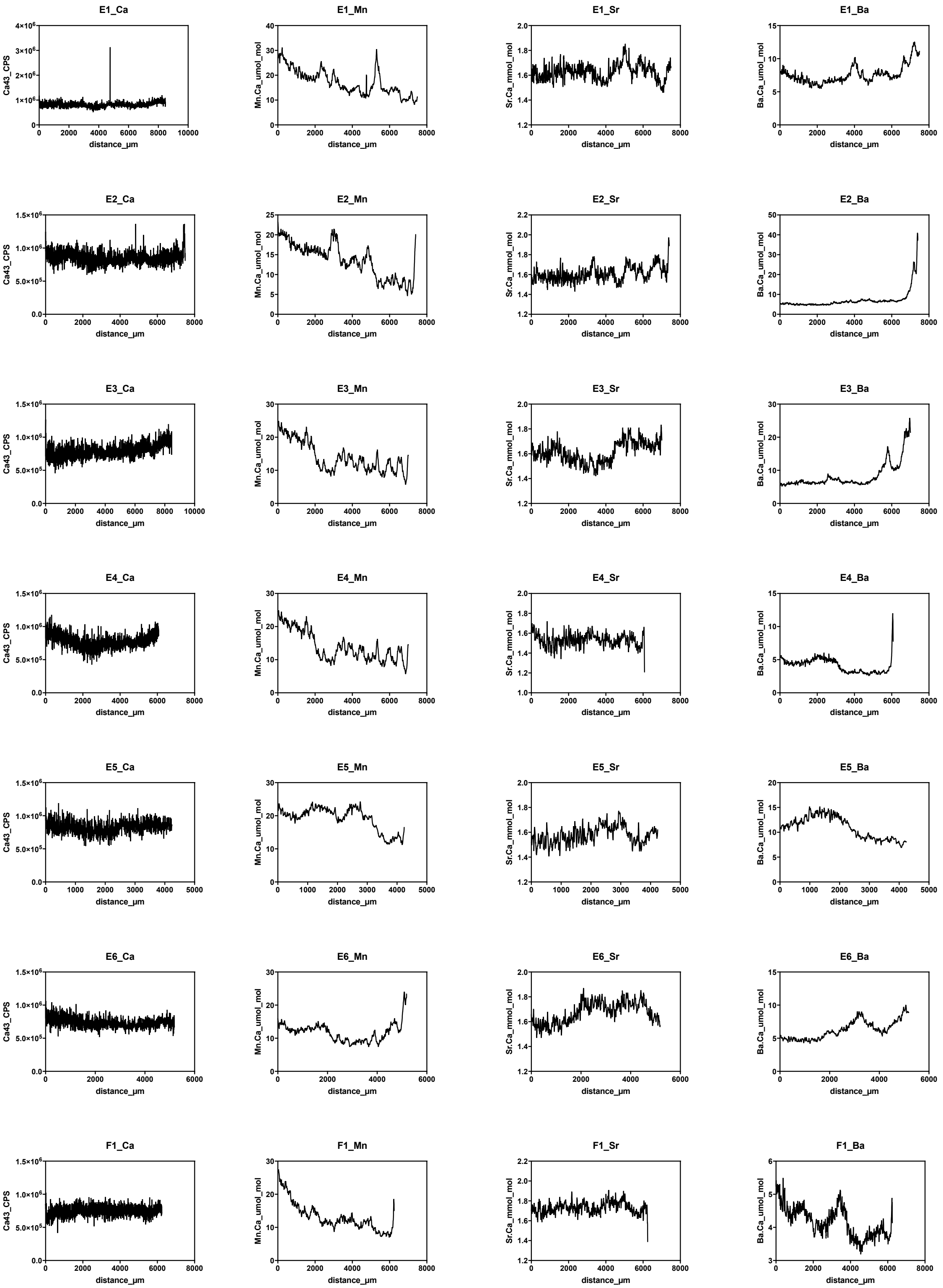

Fig S2. Continued. NOTE F5 and F6 are two replicate vertebrae from blue shark A039239

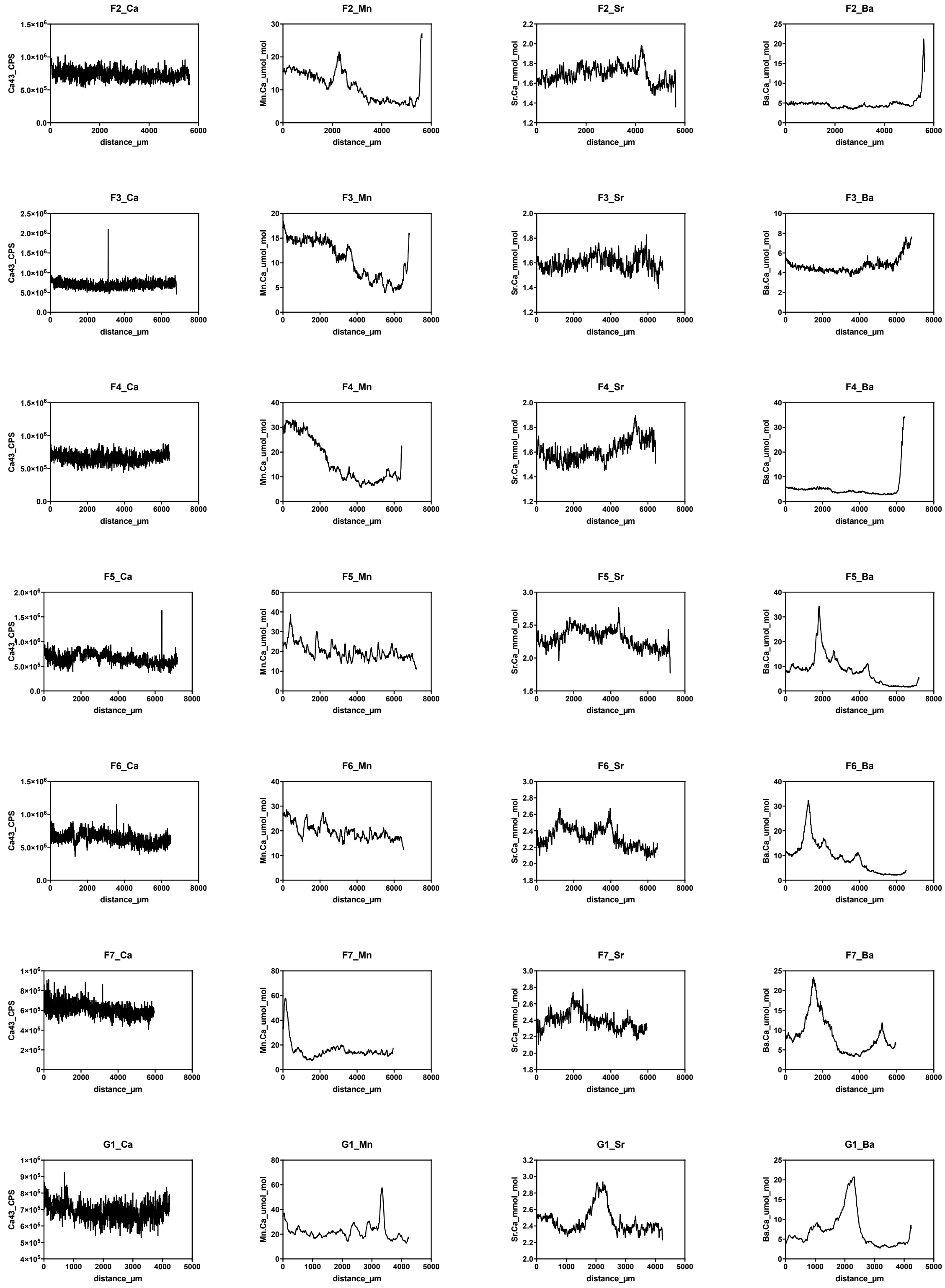

Fig S2. Continued

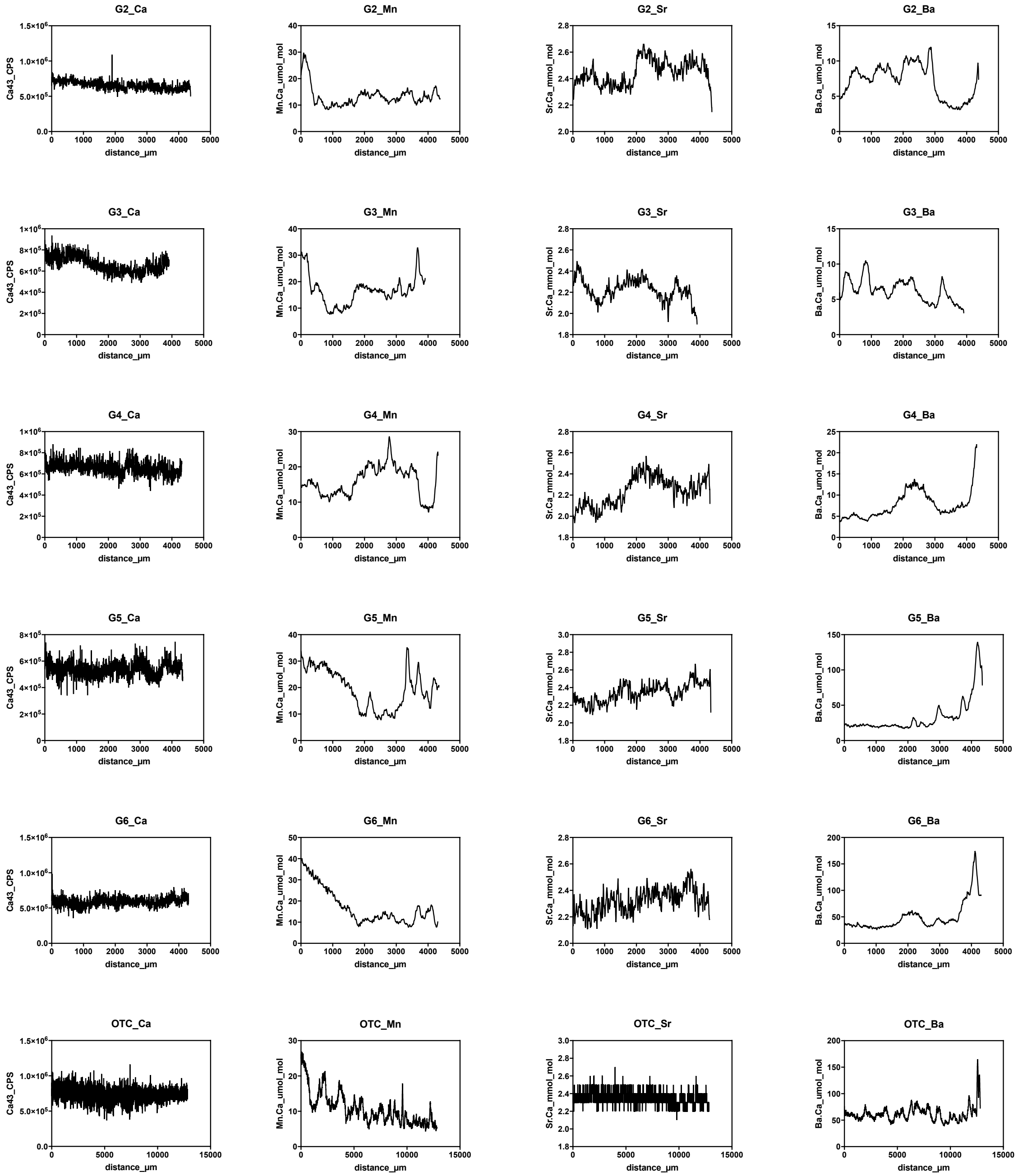

Supplement: Figures S1 and S2 [file rspb20181760supp1.pdf]
